# Clinical data for paediatric research: the Swiss approach: Proceedings of the National Symposium in Bern, Switzerland, Dec 5-6, 2019

**DOI:** 10.1186/s12919-021-00226-3

**Published:** 2021-09-20

**Authors:** Milenko Rakic, Manon Jaboyedoff, Sara Bachmann, Christoph Berger, Manuel Diezi, Philipp do Canto, Christopher B. Forrest, Urs Frey, Oliver Fuchs, Alain Gervaix, Amalia Stefani Gluecksberg, Michael Grotzer, Ulrich Heininger, Christian R. Kahlert, Daniela Kaiser, Matthias V. Kopp, Roger Lauener, Thomas J. Neuhaus, Paolo Paioni, Klara Posfay-Barbe, Gian Paolo Ramelli, Umberto Simeoni, Giacomo Simonetti, Christiane Sokollik, Ben D. Spycher, Claudia E. Kuehni

**Affiliations:** 1 Institute of Social and Preventive Medicine (ISPM), University of Bern, Mittelstrasse 43, 3012 Bern, Switzerland; 2 Service of Pediatrics, Department Women-Mother-Child, Lausanne University Hospital and University of Lausanne, Lausanne, Switzerland; 3 University of Basel Children’s Hospital Basel (UKBB), University of Basel, Basel, Switzerland; 4 University Children’s Hospital Zurich, University of Zurich, Zurich, Switzerland; 5 Public Sector Law, Zurich, Switzerland; 6 Children’s Hospital of Philadelphia, Philadelphia, PA USA; 7 Department of Paediatrics, Inselspital, Bern University Hospital, University of Bern, Bern, Switzerland; 8 Department of Woman, Child and Adolescent, Children’s Hospital, Geneva University Hospitals and Faculty of Medicine, Geneva, Switzerland; 9 Paediatric Department of Southern Switzerland, Ente Ospedaliero Cantonale, Bellinzona, Switzerland and Università della Svizzera Italiana, Lugano, Switzerland; 10 Children’s Hospital of Eastern Switzerland, St. Gallen, Switzerland; 11 Children’s Hospital of Lucerne, Cantonal Hospital Lucerne, Lucerne, Switzerland

**Keywords:** Electronic health record, Terminology harmonization, Learning health system, Paediatric research, Conference proceedings

## Abstract

**Background and purpose:**

Continuous improvement of health and healthcare system is hampered by inefficient processes of generating new evidence, particularly in the case of rare diseases and paediatrics. Currently, most evidence is generated through specific research projects, which typically require extra encounters with patients, are costly and entail long delays between the recognition of specific needs in healthcare and the generation of necessary evidence to address those needs. The Swiss Personalised Health Network (SPHN) aims to improve the use of data obtained during routine healthcare encounters by harmonizing data across Switzerland and facilitating accessibility for research. The project “Harmonising the collection of health-related data and biospecimens in paediatric hospitals throughout Switzerland (SwissPedData)” was an infrastructure development project funded by the SPHN, which aimed to identify and describe available data on child health in Switzerland and to agree on a standardised core dataset for electronic health records across all paediatric teaching hospitals. Here, we describe the results of a two-day symposium that aimed to summarise what had been achieved in the SwissPedData project, to put it in an international context, and to discuss the next steps for a sustainable future. The target audience included clinicians and researchers who produce and use health-related data on children in Switzerland.

**Key highlights:**

The symposium consisted of state-of-the-art lectures from national and international keynote speakers, workshops and plenary discussions. This manuscript summarises the talks and discussions in four sections: (I) a description of the Swiss Personalized Health Network and the results of the SwissPedData project; (II) examples of similar initiatives from other countries; (III) an overview of existing health-related datasets and projects in Switzerland; and (IV) a summary of the lessons learned and future prospective from workshops and plenary discussions.

**Implications:**

Streamlined processes linking initial collection of information during routine healthcare encounters, standardised recording of this information in electronic health records and fast accessibility for research are essential to accelerate research in child health and make it affordable. Ongoing projects prove that this is feasible in Switzerland and elsewhere. International collaboration is vital to success. The next steps include the implementation of the SwissPedData core dataset in the clinical information systems of Swiss hospitals, the use of this data to address priority research questions, and the acquisition of sustainable funding to support a slim central infrastructure and local support in each hospital. This will lay the foundation for a national paediatric learning health system in Switzerland.

## Introduction

Continuous improvements in patient care are hampered by slow and inefficient processes of generating evidence [1]. Ideally, healthcare systems should be self-correcting and self-improving: information routinely collected from patients should be directly used to improve patient care in a rapid feed-back cycle. Such systems have been described under the term ‘learning healthcare systems’ [2]. Although large amounts of patient data are collected routinely by healthcare providers, these data are typically unstructured, unstandardised, incomplete and not readily available in a format useful for research purposes. Instead, most data used for healthcare research are obtained in labour-intensive cross-sectional surveys, clinical trials and longitudinal studies that are often set up in a somewhat artificial research setting and do not fully reflect the circumstances of everyday clinical care, i.e. the “real world data”. While the results may help to improve patient care, the learning cycle is slow and costly. Each project typically involves acquiring new research grants, recruiting new staff, obtaining ethical approval, setting up the study methodology and infrastructure, recruiting participants, collecting and analysing data, writing up results and getting them published. This process can take years and generates results at a high cost. The results may be outdated by the time they are published and are potentially biased. All this limits the possibility of continuous and timely, evidence-based improvements in healthcare.

This system-wide learning process could be significantly accelerated if researchers had direct access to standardized data from the health records generated during routine healthcare, a vision shared by various initiatives in Switzerland including the Swiss Personalized Health Network (SPHN). The increasing use of electronic health records (EHRs) to document clinical and administrative information about patient’s encounters in hospitals makes such an approach technically feasible. EHRs contain information on the patients’ complaints, the clinical examination by the physician, results from investigations such as blood tests, imaging or functional tests, diagnoses and treatments. They carry the promise of a faster, cheaper and more meaningful way to evaluate and improve the quality of healthcare [2, 3]. EHRs are rarely standardized within and between institutions and data are often entered in open text fields resulting in unstructured data. These limitations could largely be circumvented if the original data were recorded in a structured and standardized way [4, 5]. A common architecture of EHRs allowing structured data capture during routine medical encounters could allow rapid analytics of healthcare data followed by fast feedback of generated knowledge, a process called a learning health system [6, 7]. The SPHN is an initiative of the Swiss Federal Government and aims to contribute to a nationwide interoperability, accessibility and use of health relevant data for evidence-based healthcare research and data-driven precision medicine [8]. SPHN follows a decentralized approach whereby standardized electronic health data remain with the healthcare provider but are shareable for research. It should allow data sharing for the purpose of data aggregation but also for distributed analysis. During the past 3 years, SPHN has set up nationwide coordinated infrastructures, prepared legal and ethical guidelines and templates to facilitate data sharing and issued calls for research projects that develop infrastructures and start using the generated data [8].

One of the infrastructure projects funded by the SPHN focused on the collection of clinical data in the paediatric setting (SwissPedData [9]), where the need for evidence-based support of treatment decisions and improvements of care is particularly important. Paediatric medicine lags behind adult medicine in this respect [10]. Evidence in paediatrics is generated slowly and at high costs because many paediatric health conditions are rare [11]. In most settings, no single research institution has enough patients to produce generalizable data that can inform clinical decisions on rare diseases. Consequently, results from studies in adults are extrapolated to children and off label use of medicines in child healthcare is common [10]. The clinical research network of Swiss Children’s hospitals, SwissPedNet [12] proposed a paediatrics-specific infrastructure development project “Harmonising the collection of health-related data and biospecimens in paediatric hospitals throughout Switzerland” (SwissPedData). The project was approved and funded by the SPHN in 2017 and was conducted during the period 2018–2020. It aimed to harmonize data and biomaterials across paediatric hospitals in Switzerland with the vision to facilitate high quality clinical research in child health. The core task of the project was to create and approve a standardized paediatric dataset for EHRs across Switzerland. Once implemented, this should facilitate paediatric multicentre research based on EHR data for healthcare, public health and causal research, taking a longitudinal life course approach.

This manuscript summarises the results of a two-day symposium with the title “Clinical data for Paediatric Research: the Swiss approach” held in Bern on December 5 and 6, 2019. The symposium was organised in the context of the SwissPedData project by the project PIs (CEK and BS) and research team (MJ and MR). The overall objective was to form a common vision for a paediatric learning health system in Switzerland. Specifically, it aimed to a) summarise the results of the project, b) describe models of paediatric learning health systems in other countries, c) give an overview of available data and successful initiatives in Switzerland, d) showcase what has been reached by SwissPedData and e) discuss prospects for the future. The target audience of the symposium included hospital-based clinician-researchers in Paediatrics, allied health professionals, and scientists engaged in clinical and epidemiological paediatric research. The program included state-of-the-art lectures by national and international keynote speakers, workshops and plenary discussions. This manuscript is structured into four section where we a) summarise presentations and discussions relating to the Swiss Personalized Health Network (SPHN) and the SwissPedData project, b) next we provide examples of parallel initiatives from other countries and c) specific existing health-relevant datasets in Switzerland d) and finally, we summarise the lessons learned and e) discussions on future opportunities from workshops and plenary discussions.

### “Harmonising the collection of health-related data and biospecimens in paediatric hospitals throughout Switzerland” (SwissPedData), an infrastructure project funded by the Swiss personalized health network (SPHN)

The presentations summarised in this section describe the aims and results of the SwissPedData project and place it in the context of the broader initiative supported by the Swiss Personalized Health Network (SPHN).

#### Building a national infrastructure for personalized health research

One of the core objectives of the SPHN is to build a national data infrastructure that will enable large scale, high-quality, national projects. *Urs Frey*, the Chair of the SPHN steering board, presented the vision and guiding principles, the practical implementation and the current status of this initiative. The vision is that future researchers can submit a query through a central portal to quickly identify a specific cohort from among all patients at university hospitals in Switzerland and, provided the legal and ethical requirements are met, can access and analyse data from these patients in a project-specific space in a secure computing environment. In reaching this objective SPHN follows a decentralized approach whereby the data remain at the individual hospitals, but are shared on a project-specific basis through a secure network maintained by SPHN Data Coordination Centre (DCC). The SPHN is committed to the FAIR principle, whereby patient data must be Findable, Accessible, Interoperable, and Reusable. The practical implementation requires key changes at the technical and governance level in the different phases of the data flow such as finding, requesting, receiving and using the data. A federated query system and data catalogue will be implemented to allow the quick and reliable estimation of availability of data and samples for a specific research project. On the data-provider side, governance processes and compatible IT infrastructure solutions need to be in place to allow extraction and pseudonymization and ensure semantic interoperability for data exchange. Standards and templates for Common Data Transfer and Use Agreements (DTUA) are being developed to facilitate data exchange and define key regulatory requirements regarding the rights, responsibilities and obligations of all parties involved.

Much has already been achieved. Through various infrastructure implementation and development projects SPHN has developed standards, procedures and technical infrastructure to enable the nationwide interoperability and exchange of molecular and clinical patient data. Examples of such infrastructure development projects are E-General Consent, which aims to develop a nationwide harmonized interactive electronic general consent to use data generated in hospitals for research, Swiss BioRef, which aims to generate accurate individualized reference ranges for results of blood tests, and SwissPK^cdw^, which aims to optimize paediatric dosing regimens by applying pharmacokinetic modelling to data from routine drug monitoring in clinical data warehouses [13]. In parallel, so-called driver projects started to use the data generated in the warehouses to answer specific research questions. Examples are a) the Personalized Swiss Sepsis Study, which uses machine learning to analyse continuous intensive care unit (ICU) monitoring, laboratory, microbiology, and -omics data for personalized sepsis management, b) IMAGINE, which proposes to build a Swiss-wide infrastructure for image-based biomarker research & analysis, and c) The Swiss Heart Failure Network that will create a standardized data infrastructure for a Swiss Heart Failure Registry and integrate multi-dimensional features from patient data into machine learning-based diagnostic and risk scores [14]. Throughout the SPHN processes, a number of general gaps and challenges have been identified including specific organizational, legal and regulatory aspects of data transfer between hospitals, the need for more patient involvement, for specific concepts for governance, processes and infrastructures for -omics data, and for a strategy for engagement with the industry and international consortia.

**Fig. 1:**
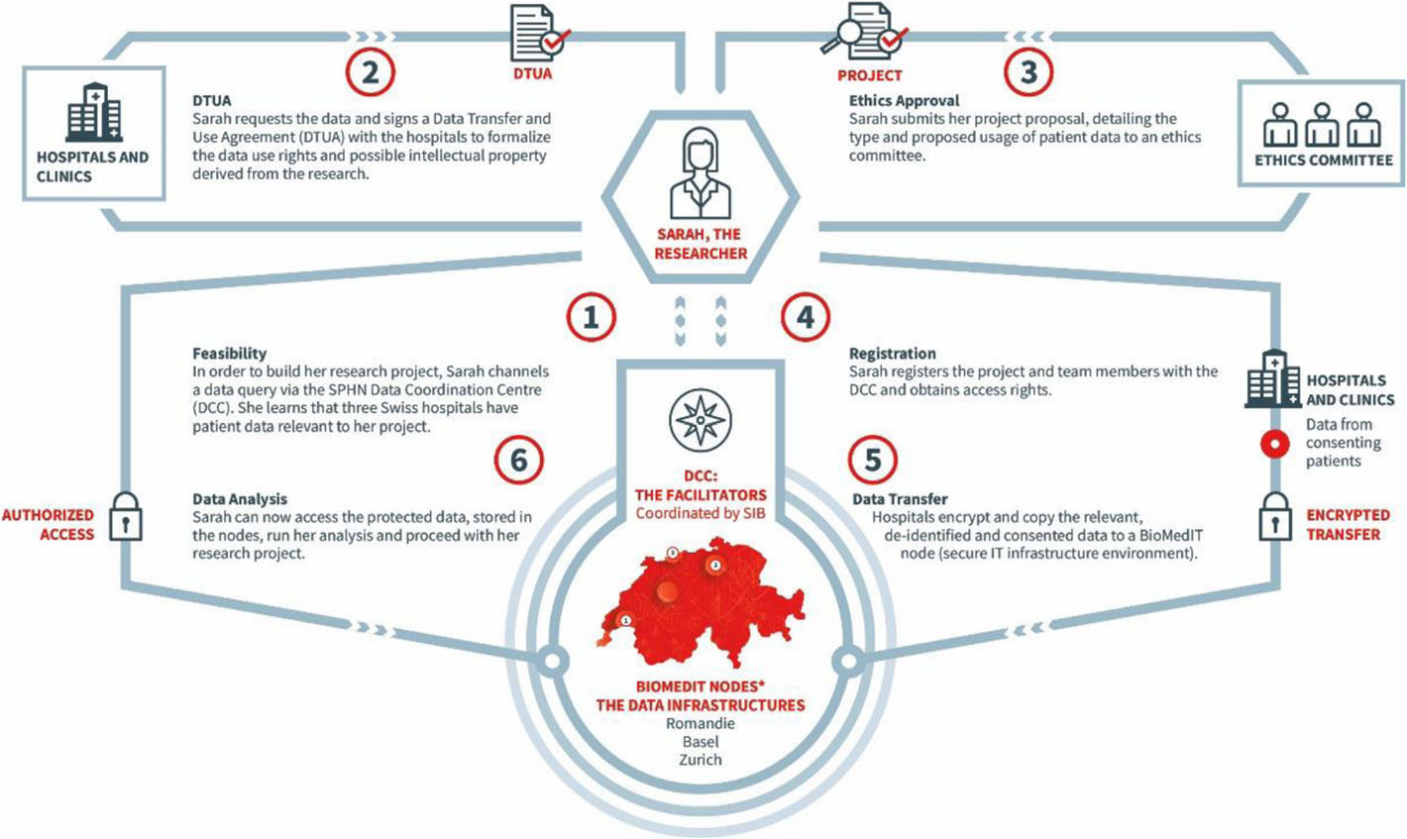
The researcher’s new path to nationwide clinical data ([16], page 5)

*Katrin Crameri*, Director of the Swiss Institute of Bioinformatics’ (SIB) Personalized Health Informatics Group, which manages the SPHN Data Coordination Center and the Biomedical Information Technology (BioMedIT) Network Project [15], provided details about the organisation of the infrastructure network and how researchers will be able to set up a specific project (Fig. 1). A secure IT infrastructure for high performance computing and processing of sensitive, biomedical data has been set up through a network of three interoperable BioMedIT nodes, one in Lausanne (operated by SIB), one in Basel (operated by the University of Basel), and one in Zurich, operated by Swiss Federal Institute of Technology Zurich (ETHZ). The BioMedIT portal is the single entry point for registered researchers providing access to the network, which runs under one common Information Security Policy. If the data sharing and data usage requirements for a specific project are in place (patient consent, ethical approval, DTUA), the patient data can be assembled from the five university hospitals’ Clinical Data Warehouses, biobanks, −omics platforms, cohort studies, or other data sources. Data packages for a specific project are transferred (end-to-end encrypted) to an isolated project space on one of the BioMedIT nodes for the researcher to access. Within the project space, BioMedIT provides researchers with cutting edge technology for cloud computing, big data storage and high-performance computing.

#### Achieving data interoperability

The specific challenges of reaching interoperability for laboratory data were highlighted by *Alexander Leichtle*, who is the principal investigator (PI) of the SPHN infrastructure development project Swiss BioRef, which aims to build a nation-wide resource for generating personalized reference ranges for laboratory tests. These data come from numerous different sources in heterogeneous formats. Moreover, some data types like microbiology data are difficult to map. *Leichtle* highlighted the fact that when working with routine data, continuous monitoring tools must be in place. Some Swiss hospitals already implemented the use of LOINC codes [17] for laboratory data. The 2018–2020 eHealth strategy takes this issue into account and recommends that semantic standards should be used whenever possible.

Ensuring interoperability of clinical data is a particular challenge as both *Crameri* and *Leichtle* highlighted. These data are typically not standardized and often highly unstructured. In principle, two complementary approaches are followed by the SPHN. The first one is to harmonize content and format of clinical data as it is collected or entered into a clinical information system. This approach is being followed by specific SPHN projects such as the paediatric SPHN project “Harmonising the collection of health-related data and biospecimens in paediatric hospitals throughout Switzerland”. The other approach is to map already collected data into a common data model. A common data model specifies the structure and standards for a set of data domains. For unstructured data Natural Language Processing may be necessary to structure the data. The Clinical Data Semantic Interoperability Working Group of the DDC is leading the process of defining common data models within the SPHN. This includes a core dataset of basic data on all patients, which is devoted to support distributed queries and an extended dataset covering the needs of specific projects.

#### The aspects of ethical, legal and social implications (ELSI) - the particular case of minors

The SPHN recognizes that working toward efficient production of scientific knowledge as a societal good must go hand in hand with promoting the rights, interests and well-being of the research participants and generating public trust. The SPHN has therefore produced ethical guidelines built on four general principles: respect for persons, privacy, data fairness and accountability [18].

*Philipp do Canto*, an expert of public health law and legal advisor to the SPHN project SwissPedData, gave an overview on ethical and legal aspects specifically related to collection and use for research of health-related data and bio-specimens from minors. Under Swiss law, minors (children < 14 years and adolescents < 18 years old) are considered vulnerable persons. For these groups, specific requirements apply in addition to general requirements for human research on adults. In particular, the Swiss constitution requires the principle of subsidiarity, under which research that involves minors is only legitimate if findings of equal scientific value cannot be achieved with adults. Furthermore, only projects that impose minimal risks and burden on participants and that are expected to produce substantial findings beneficial for people with the same problem may be conducted on children (with or without capacity of judgement) or on adolescents without capacity of judgement. However, this should not prevent minors from being included in studies as they also have the right to receive evidence-based medicine tailored to their age group. Further specific requirements mainly concern principles for consent. Minors must be involved as far as possible in consent giving, with increasing involvement as they grow older. If it is unclear whether a minor has the capacity of judgement, the latter must be presumed. All minors must be given the possibility to revoke consent after reaching maturity. The decentralized political system of Switzerland with 26 cantonal data protection acts greatly complicates the exchange of data. Specific contracts between institutions have to be adapted for each situation. Detailed legal standards for biobanks and big data applications are still lacking. *Do Canto* emphasized, that while studies must comply with legal standards, the legal standards themselves should be adapted to societal and technological developments for the benefit of patients.

#### SwissPedNet, the Swiss research network of clinical Paediatric hubs

*Klara Posfay-Barbe*, current president of SwissPedNet, explained how the network was founded in 2012 by the five university children’s hospitals in Switzerland (Basel, Bern, Geneva, Lausanne and Zurich) and the three paediatric teaching hospitals in Aarau, Lucerne, and St. Gallen [12]. In 2015 the department of paediatrics of the Italian speaking canton Ticino joined as a ninth clinical hub. All nine hubs provide age-specific infrastructures, paediatric-trained personnel, paediatric guidance documents and specialized quality management systems for clinical trials in children. SwissPedNet promotes clinical research in paediatrics by supporting paediatric clinician-scientists in their commitment to high-quality clinical research, by facilitating national and international collaboration, providing training opportunities for young researchers and increasing acceptance of clinical trials in children. It promotes, facilitates, coordinates and conducts clinical trials in children, from newborns to adolescents, in all paediatric disciplines. SwissPedNet has two associated technology platforms: SwissPedPha, which provides services in paediatric pharmacology, and SwissPedRegistry, which coordinates national registries and cohort studies and advises on the conduct of registries and observational studies in children. SwissPedNet receives direct federal funding to support the infrastructure of the nine clinical hubs, SwissPedPha and SwissPedRegistry. It broadly shares the vision of the Swiss Personalized Health Networks and contributes to its developments with a Paediatric focus.

#### SwissPedData *-* Harmonising the collection of health-related data and biospecimens in paediatric hospitals throughout Switzerland

**Fig. 2:**
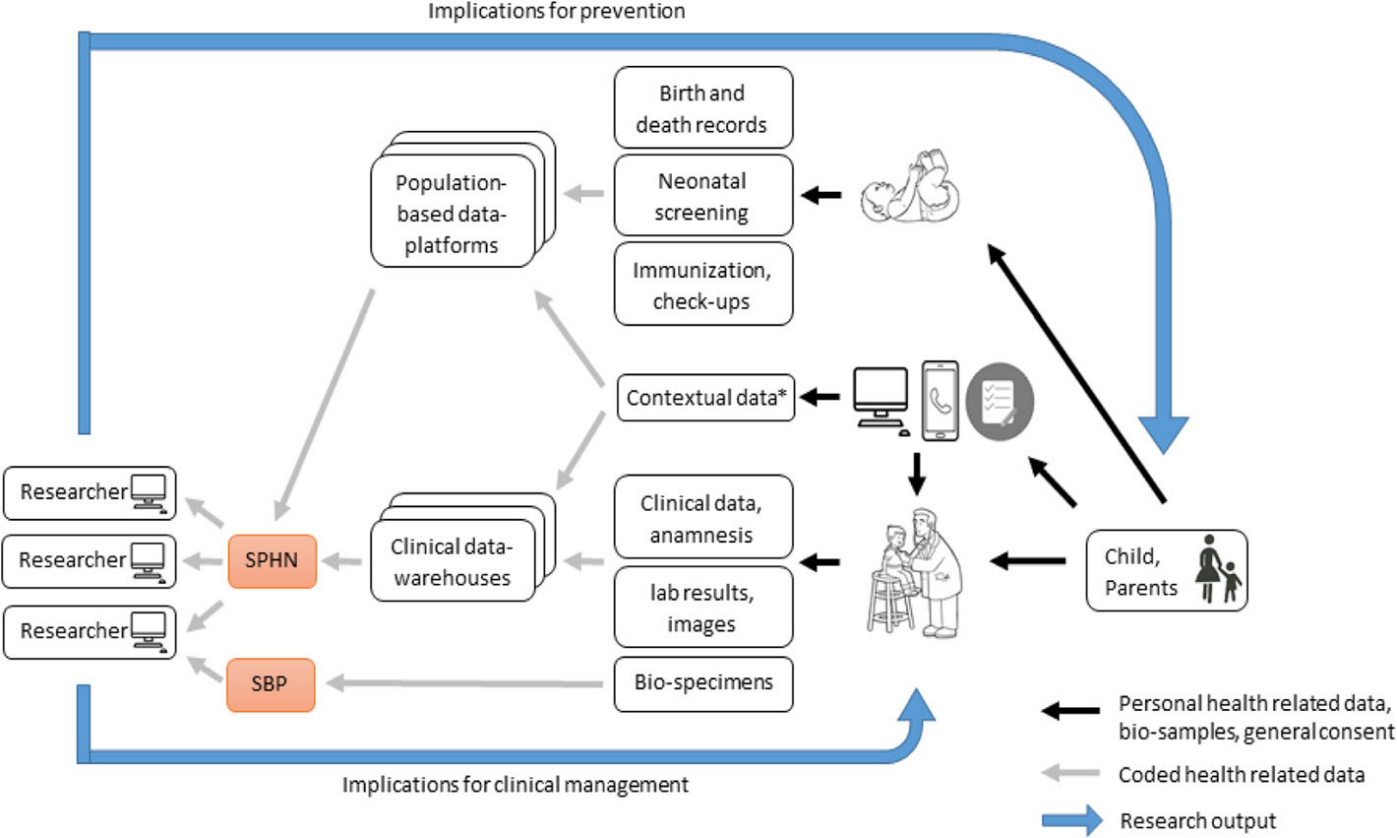
Schematic outline of the future use of clinical and population-based data relevant for child health for paediatric research in Switzerland, as proposed in the project SwissPedData (own illustration)

*Claudia Kuehni*, primary investigator of SwissPedData, gave an overview about this SPHN infrastructure development project, which was funded in 2017 and ran from 2018 to 2020. SwissPedData’s goal was to develop an infrastructure for affordable, high-quality prospective clinical data on every child in Switzerland. Its three work packages (WP) aimed, 1) to create an inventory of resources and ongoing research with routine data on child health in Switzerland; 2) to develop a national standardized dataset for children visiting paediatric hospitals; and 3) to evaluate whether parents would be willing to actively contribute information on their child’s health to SwissPedData (Fig. 2).

**The inventory of work package 1** has been completed and consists of routine data collected by the Swiss Federal Statistical Office, such as birth, cause of death and hospital episode statistics, and of non-health related contextual data such as migration statistics and census data. Limited datasets are available from the primary care setting and specific initiatives, such as the Swiss electronic vaccination record [19]. In addition, numerous observational studies and registries collect data on specific diseases. Some of these resources were discussed during the symposium and are summarised in section 3 of this paper.

**Work package 2 was the core element** of the project and involved an extended Delphi process with all paediatric hospitals and representatives from almost all paediatric subspecialties in Switzerland. We involved, from all hospitals, senior consultants with expert knowledge, as well as junior physicians who enter the data into the hospital information system and use it for daily clinical work. The dataset that was developed in this effort includes a core module with general paediatric information that is relevant for all patients, and subspecialty modules that are relevant for subsets of patients. It is compatible with international datasets, in particular the dataset from PEDSnet (chapter 3), but has also additional variables that are relevant for the Swiss context or for specific specialties or research interests. Version 1 of SwissPedData has been finalised and is ready for implementation into the clinic information software of participating hospitals. While this takes place, efforts will need to be taken to secure sustainable funding for local support in each hospital, and for central core structures. Based on templates from the SPHN, DTUA are being developed for use by all hospitals.

**Work package 3** aimed to investigate, whether parents are willing to provide additional information into the hospital information systems, which can be used for clinical care and for research. This is particularly important for information that is relevant for child health and included when physicians take a complete medical history, but is often not documented consistently in medical records due to time constraints, particularly in acute care: family history, environmental exposures and lifestyle such as active and passive smoking or physical exercise, socioeconomic information and data on social contacts, education and schooling. Work package 3 aimed to pilot paper-based and electronic questionnaires of different length that could be completed by parents in the case of in- or outpatient visits. Evaluation of such parental questionnaires for respiratory outpatients is ongoing as part of the SPAC Study [20]. Together, standardized electronic patient records and parental questionnaires should in future boost hospital-based paediatric clinical research in Switzerland.

### The international landscape - parallel initiatives in other countries

The symposium opened a view on the international landscape, with three examples of how data obtained during clinical care can feed into research, which in turn allows to improve healthcare. These included the PEDSnet initiative in the US, the use of linked clinical inpatient data for observational research and nested trials in the UK, and a multicenter electronic management tool for asthma care in the Netherlands.

#### PEDSnet: a learning health system for Paediatrics

**Fig. 3:**
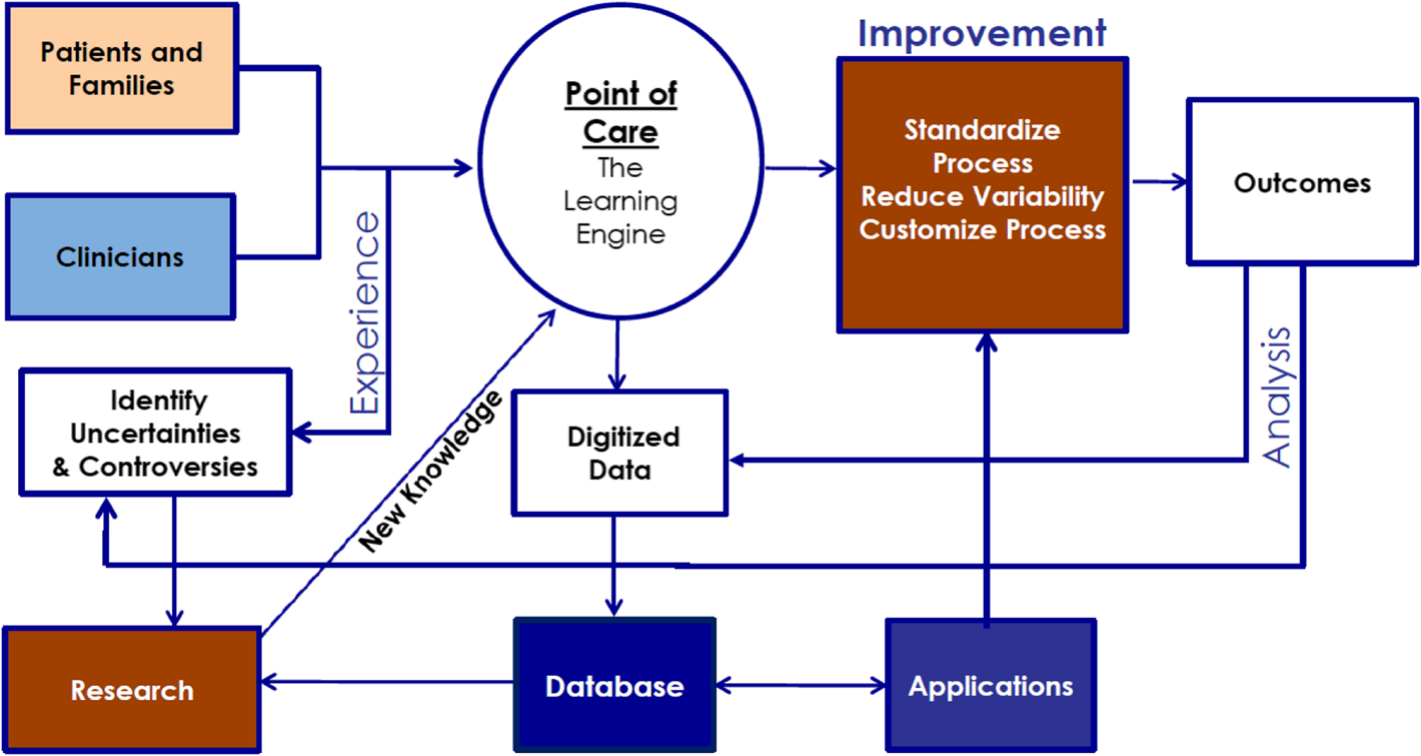
Improving outcomes with a learning healthcare system: PEDSnet model (by Christopher Forrest)

*Christopher Forrest*, principal investigator of the US PEDSnet initiative [21], discussed different aspects of PEDSnet. A first talk, entitled “From concept to prototype to national scaling” focused on history, organization, and governance. PEDSnet is a national Paediatric Learning Health System that was founded in the U.S. in 2014 by eight U.S. Children’s Hospitals [22, 23]. It was motivated by the need to obtain child-specific data on the effectiveness and safety, particularly long-term events, of new and already approved drugs and other types of medical, surgical, and behavioral interventions. Complementary to expensive clinical trials in small groups of selected patients, use of electronic health record data on a large-scale has potential to improve the sparse evidence base. The overall objectives of PEDSnet are to scale-up a collaboration platform that enables rapid improvement in the outcomes of ill children and of the health systems that serve them. Instead of the traditional research cycle, which can take 15 years from the formulation of the research idea to the implementation of the findings into healthcare, PEDSnet aims to be a learning health system (LHS) where clinical care, information technology and research are closely interlinked, research is done at the point of care and feeds directly back into internal improvements within a short time scale (Fig. 3).

The Steering Board and committees for research, data and engagement include senior representatives from every participating Children’s Hospital. Other stakeholders closely involved in the organization of PEDSnet are healthcare organizations, researchers, clinicians, patients and families. Currently, PEDSnet hosts analysis-ready standardised longitudinal data from primary, secondary and tertiary care for over 6.8 million patients.

Key characteristics of the PEDSnet LHS are, (1) that it uses a common interoperable, prospectively designed platform, (2) builds trust among stakeholders, (3) optimizes electronic health records to support that data are entered once, but are used many times – for care, improvement, registries, and research, (4) continuously expands and strengthens its scalable infrastructure, (5) links electronic health record data with other data sources, and (6) secures a sustainable future.

In his second talk, *Christopher Forrest* gave specific examples and case studies, to show how PEDSnet conducts research based on data from routine care, and how results are fed back to improve healthcare in participating hospital**.** PEDSnet allows to conduct different types of studies:

- **Pragmatic clinical trials:** the PEDSnet infrastructure can be leveraged for prospective clinical research, including studies that involve collecting data directly from patients in cohort studies or clinical trials. The data network is used to identify specific patients who meet eligibility criteria for a study. Patients’ encrypted identifiers are then sent to each local institution, where a reidentification process is undertaken before chart review to ensure that they meet the selection criteria. Examples are the BACK-OFF trial in Juvenile Spondyloarthritis [24] or a surgical trial comparing three approaches to treat kidney stones [25].
- **Pseudo-trials:** This type of design was used to show the superiority of infliximab versus standard care for Crohn’s disease in patients with new-onset moderate or severe episodes of illness. Infliximab in Crohn’s disease had previously been prioritized over standard of care in paediatrics following positive results in adults, although no RCT was ever conducted in children. Conducting a RCT today would not have been acceptable from both a clinical and ethical perspective. In collaboration with the learning network ImproveCareNow [26], PEDSnet conducted a “pseudo-trial” study that used available data and compared episodes of care that used infliximab with episodes with standard treatment [27].
- **Descriptive epidemiology:** The prevalence of obesity and overweight in 2–17 year olds was similar in EHR-derived data from PEDSnet (18 and 17% respectively) and in the National Health and Nutrition Examination Survey (NHANES; 18 and 16%). PEDSnet estimates were more stable because the dataset was much larger [28].
- **Computable phenotyping**: PEDSnet data is used to develop and evaluate computable phenotypes for the identification of patients with rare diseases, such as cancer and glomerular disorders [29, 30].
- **Longitudinal observational studies:** PEDSnet data provided quantitative evidence of the effect of obesity on incident asthma (30% increase in risk [31]), of an association between antibiotics use before 2 years of age and body mass index at age 5 [28, 32, 33] and of an association between oral thrush by age 1 year and development of childhood caries at the age of 2 to 5 years [34].
- **Comparative effectiveness study:** Gastric banding was shown to be inferior regarding reduction of body mass index compared to gastric bypass and sleeve gastrectomy in youth [35].
- **Covid-19:** A recent example is that PEDSnet used paediatric patients’ EHRs to describe the epidemiology of infected patients in the United States [36].

The third talk focused on the PEDSnet data network, its common data model, the system architecture, data flows, and the data quality program. Data quality was an overarching concern during the design of the PEDSnet data network. The goal was to create data of high quality, which are easily accessible. PEDSnet has three relevant teams of data experts. The data committee oversees the data model and leads the strategic development of network. The data coordinating centre updates the structure and semantics of the data model and network (the pipeline operations), automates the cross checks to maintain high quality, and provides guidance on how to use complex data to improve research. The centre specific teams extract, transform and upload the data and give site level guidance and implementation. Every 3 months, new data are uploaded and crosschecks are done to test the validity and assure high quality. This is automated by staff with a background in health research and informatics and the tools are publicly available for use in R.

#### Experiences from linking routine hospital datasets in the UK, and their use for observational studies and nested clinical trials

*Katie Harron*, from the University College London, showed how administrative data, including routine hospital data, can be used to support clinical trials, for instance by capturing outcome information or helping to assess the generalisability of results. In one study, long term educational outcomes on the participants of seven historical infant formula trials were extracted from administrative education records [37]. In another example, routine datasets from paediatric intensive care units (PICUs) and laboratory surveillance were used to generalise the results from a large randomised clinical trial on the use of antibiotic-impregnated central venous catheters to prevent bloodstream infections in PICUs [38]. These examples rely strongly on the ability to match the records that belong to the same person from different datasets (record linkage). This can be done in a deterministic manner, which requires an exact match on one (e.g. social security number) or more key identifiers contained in both datasets, or in a probabilistic record linkage approach, which allows for some degree of mismatch in the common variables caused by errors or omissions during data entry [39]. Linkage errors including false matches (records of different persons assigned to the same person) or missed matches (records of the same person assigned to different IDs) can bias the results of a study. This can, for instance, occur through the misclassification of outcomes, over- or undercounting of individuals and by excluding unlinked cases. It is therefore important to assess the quality of data linkage and the potential for biases [40]. *Katie Harron* highlighted several ways in which this can be done, for instance, through the use of positive and negative controls, comparisons with external references and comparison of characteristics of linked and unlinked records [41].

#### Lessons learned from online paediatric asthma management

*Peter Merkus,* from the Radboud University Medical centre (UMC) Amalia Children’s Hospital in Nijmegen, Netherlands, presented the Luchtbrug® (air lift) study, which uses an online platform for the medical management of children with asthma, which enables patients and their parents to monitor their disease and communicate directly with paediatricians. Between 2011 and 2014, children with asthma were randomized to usual care with clinical visits every four months or to a novel management approach with clinical visits every eight months plus monthly online asthma monitoring encounters. Children reported their asthma symptoms and medications to the online platform, which was checked by a respiratory specialist nurse. The nurse gave feedback to patients and informed clinicians in case of asthma exacerbations. The online management program reduced outpatient visits by 50% while maintaining asthma control. There was no difference in asthma exacerbations, hospital admissions, and daily medication doses between the two groups [42, 43]. The Luchtbrug® study serves as a new concept how patients with a chronic disease can contribute to a clinical data platform. In a follow-up study, Luchtbrug Connect®, self-reported data is complemented by results on pulmonary function from smart inhalers in selected patients with poor adherence, low symptom perception or severe asthma.

Lessons learnt from the study are: to develop the tool from the patient’s perspective; to aim for sustainable implementation from the start and anticipate on what is needed; to involve patients and patient organisations in developing and improving the platform; to create obvious personal benefits for patients, aiming for a WIN – WIN situation; to address barriers for implementation (financial and interoperability) adequately; to provide convincing evidence for added value and cost-effectiveness to health insurance companies, hospitals and governments; to invest in a team with common ambition; to ask for professional help in particular with relation to privacy, information technology, legal contracts and similar; to get out of the comfort zone as a researcher and connect with the national governmental institutions and bodies; and, finally, to expect that the processes will take some time [44].

### The current landscape in Switzerland: overview of available routine data and examples of successful research based on real world, standardized healthcare data

The symposium included a series of presentations highlighting successful examples and potential future usages of routine datasets for clinical research in Switzerland. The case studies spanned a variety of information resources including national administrative datasets collected by the Swiss Federal statistical office, expert (Orphanet) and surveillance (Swissnoso) networks, registries (NeoNet, Swiss Childhood Cancer Registry), clinical cohorts (Swiss Paediatric Airway Cohort) and primary care electronic medical records (FIRE project).

#### Routine administrative data collected in the Swiss Federal Statistical Office and how they can be used for research

*Adrian Spoerri*, head of SwissRDL – Medical Registry and Data Linkage, a centre of excellence for registries at the University of Bern – gave an overview of health-related administrative datasets available at the Federal Statistical Office (FSO) and the possibilities for record linkage with clinical datasets, cohort studies or registries for research purposes. He emphasized that researchers should attempt to make use of the available administrative datasets. Record linkage of these datasets with each other and with other datasets for research purposes has a firm legal basis and is often technically feasible. Such record linkages are generally performed by the FSO and detailed instructions and an application form are available from the FSO website [45]. For instance, a researcher might want to use the Hospital episode statistics (HES) to identify multiple hospitalisations or hospitalisations in another hospital for a given set of patients. The HES has collected sociodemographic variables, characteristics of hospitalizations, diagnoses and interventions on all patients from all hospitals and birth centres since 1998. An anonymous code allows tracking multiple hospitalizations from the same patient and linkage of different statistics within the FSO. As another example, a researcher may want to use national mortality records to update vital status in a clinical cohort study. Such a linkage requires re-identification of a social security number based on personal identifying information (PII) such as names, dates of birth, and address. After linkage, the linked data can only be used to answer the specific research question and must be destroyed and cannot be used to update the cohort dataset permanently. In the future, the establishment of linkage centres might greatly facilitate the linkage of personal data. In these centres, the data from different sources are linked using PII and the linked data (without PII) is then made accessible to researchers in a data hub via secure remote access. A successful example of how record linkages between multiple administrative datasets can be used for health research is the Swiss National Cohort study (SNC) [46]. The SNC is research platform linking national censuses since 1990 with each other and with national datasets on migration and mortality, and has led to a large body of peer-reviewed publications.

#### Orphanet, a tool for healthcare and research on rare diseases

*Loredana D’Amato Sizonenko,* coordinator of Orphanet Switzerland [47], gave an overview of the resources provided by Orphanet to improve the visibility of rare diseases, provide high-quality information and expertise on rare diseases and contribute to generate knowledge on rare diseases. Orphanet Switzerland is a partner of the International ORPHANET Network, the reference knowledge base for information on rare diseases and orphan drugs. Orphanet has created a nomenclature for rare diseases which allows assigning each disease to a unique and stable ORPHA-code, which is cross-referenced to other international nomenclatures and translated into nine languages. It is multi-dimensional and multi-hierarchical. ORPHA-codes allow for semantic interoperability of rare disease diagnosis, in health-information systems but also in research. In Switzerland, some hospitals already embed ORPHA-codes in their clinical information system in order to allow for quick identification of patients with a rare disease.

#### Research based on routinely obtained data from paediatric hospitals in Switzerland

Research projects in different paediatric disciplines already use data generated in routine care and harmonized on a national level for health-care research. This includes registries and cohort studies in paediatric oncology, neonatology and infectious diseases (using data from inpatients) and paediatric pulmonology (using data from outpatient encounters).

*Mark Adams*, coordinator of SwissNeoNet, presented this learning health system for neonatology in Switzerland. SwissNeoNet [48] is a population-based registry hosting the official medical quality register for the neonatal intensive care units (9 units) and intermediate care units (11 units). SwissNeoNet aims to maintain and improve medical care for high-risk newborns and their families. A minimal dataset is collected for all Swiss infants born below 34 weeks of gestational age, those with a birth weight of less than 1500 g and those with neonatal asphyxia. Follow-up data are collected after 2 and 5 years in 16 neuro-developmental paediatric units for infants below 28 weeks gestation and those with asphyxia. Data are either imported automatically from the clinic information system in SwissNeoNet or entered by the units through a web form. All data are checked for plausibility and reliability. Suspicious entries have to be corrected by the units. *Mark Adams* explained that participation in SwissNeoNet has several advantages for the units: first it provides a data repository, where units can extract their own data, combine them with follow-up data on a local level and use them for local research. Analysis on a national level needs agreement by all participating units. Second, the project performs regular unit-to-unit comparison and thus provides the possibility of quality control and national and international benchmarking. Results are used for quality improvements. Third, the collected data allow the comparison of outcome data between Switzerland and other countries [49].

*Rami Sommerstein*, Head of Research & Development at Swissnoso, presented Swissnoso, a research program aiming to reduce infections in hospitals and long-term care facilities in Switzerland [50]. Swissnoso acts as a national non-profit association of experts in hospital hygiene and develops research projects and guidelines aiming to reduce healthcare-associated infections in hospitals or long-term care facilities. The national program “Swiss Clean Care” includes several modules, which support hospitals in the prevention of healthcare-associated infections. The Surgical Sites Infection (SSI) Surveillance module monitors the frequency of postoperative wound infections, with 174 clinics contributing data [51]. The SSI Intervention module aims to reduce post-operative wound infection rates by implementing three interventions: correct hair removal, preoperative skin disinfection and antibiotic prophylaxis. When these measures are adhered to in 90% of cases, the wound infection rate may be reduced by at least 10%. The “CleanHands” module allows electronic recording of hand hygiene adherence in health care workers by using a mobile device during observations of hand hygiene. The tool provides automated analysis and national benchmarking that will allow immediate feedback of the results. Currently, over 90 clinics are using the instrument. For the project StAR (Strategy against Antibiotic Resistance), Swissnoso develops together with the Swiss Society for Infectious Diseases SSI and the Swiss Society for Microbiology SGM prescription guidelines for antibiotics, guidelines for the prevention and control of outbreaks, and a concept for the proper handling of antibiotics.

*Ben Spycher*, head of the research group Environmental and Spatial Epidemiology at the ISPM Bern, described how data from the Swiss Childhood Cancer Registry can be combined with various other datasets including clinical and administrative data for research purposes. The Swiss Childhood Cancer Registry (formerly SCCR, as of 2020 Childhood Cancer Registry, ChCR, [52]) was founded in 1976 and is a population-based registry with nationwide coverage including all children and adolescents under the age of 21 who are diagnosed with cancer in Switzerland. Data completeness has been more than 95% since 1995 for children between 0 and 15 years of age. Vital status is updated through a combination of passive and active follow-up and record linkage with the mortality records The ChCR forms the basis of the Swiss Childhood Cancer Survivor Study (SCCSS), which collects long-term follow-up information from 5-year survivors through questionnaires and allows investigating a wide range of somatic and psychosocial outcomes including second primary malignancies and late effect of cancer treatments, educational achievement, and health-related quality of life [53]. Furthermore, probabilistic record linkages of the ChCR records with the SNC allowed to investigate the spatial distribution of a range of potential risk factors of cancer incidence in children including natural background radiation and proximity of residence to highways and to nuclear power plants [54–57].

The Swiss Paediatric Airway Cohort (SPAC) was presented by *Carmen de Jong*. SPAC is a national longitudinal study using data from outpatient encounters for respiratory problems in paediatric hospitals, i.e. children seen in respiratory outpatient clinics [20]. SPAC is an observational multipurpose cohort study embedded in routine care, which aims to describe the spectrum of respiratory problems leading to outpatient visits; to distinguish clinical phenotypes of wheeze and cough [58]; to describe prognosis and predict the long term course of respiratory disease, and to investigate, compare and harmonise diagnostic practices, treatment strategies, and preventive measures used in healthcare in Switzerland [59]. SPAC serves also as a sampling frame for recruiting children for nested studies. Data are collected from medical records and via questionnaires to parents. Seven hospitals and 2 private practices have invited over 3000 patients in 28 months. The overall participation rate is 63%, but varying between clinics from 48 to 89%. In order to increase the participation rate, local conditions need be taken into account during recruitment so that it corresponds to routine care procedures. For example, pro-active monitoring and regular reminders, especially in the beginning, improve participation rate. SPAC shows that conduction of a study embedded in routine care but involving active contributions for participants is feasible in Paediatric Outpatient care, achieves relatively high response rates, and can run almost automatically after procedures are well established.

#### Research based on data obtained in primary Care in Switzerland

Obtaining standardized digital data from primary care poses a particular challenge. *Sven Streit*, Associate Professor at the Institute of Primary Healthcare (BIHAM), University of Bern, presented the FIRE project (Family medicine ICPC Research using Electronic medical records [60]), a database currently containing primary care data of over 862′990 patients from more than 500 general practitioners (GPs). Data collected within FIRE include vital parameters, date of consultation, age, gender, medications, diagnoses, and laboratory values. Data are anonymous and automatically transferred every 2 months to the data server hosted at the University of Zurich. This dataset can be used for cross-sectional studies, for the recruitment and follow-up of cohorts, but also to embed new clinical trials.

#### Workshops

The symposium included two workshops in small groups of heterogeneous composition including junior and senior participants, clinicians and non-clinicians. Each group was asked to (1) brainstorm and collect clinical questions, where evidence is lacking or contradictory; (2) sketch a short research proposal describing the type of data needed and the most appropriate study design; (3) discuss the feasibility of their research project, including sample size and possibility of recruitment; and (4) suggest suitable funding for the hypothetical proposal. The underlying aim of the exercise was to raise awareness about uses of a standardized national health record dataset for research and healthcare.

The short research proposals spanned a broad range of medical topics and study designs. Examples included a diagnostic study on the validity of the tests used for auditory screening in newborns; a benchmarking study assessing quality of treatment for bronchiolitis across different Children’s hospitals; a cohort study on the incidence of hearing loss after treatment with aminoglycosides in infancy; a cohort study on kidney injury after treatment with acyclovir; and a randomized clinical trial comparing the effectiveness of different treatment regimens for type 1 diabetes. Several of the proposed studies aimed to complement the hospital dataset with available data from other sources such as the federal statistical office, or with the collection of additional data through questionnaires or specific examinations. Strengths and weaknesses of the proposed projects and aspects of implementation and feasibility were then critically discussed in the plenum. Overall, this exercise gave a first impression of the possibilities that will open and did whet the appetite for more.

#### Implications, sustainability and steps forward

The plenary discussions during the symposium and at its end focused on the next steps in the process of setting up a paediatric learning health system in Switzerland.

#### Implementation of SwissPedData in the electronic health records of Paediatric teaching hospitals

Implementation of SwissPedData, Version 1, into the electronic health records of all participating children’s hospitals is the obvious next step in the process. This will take some time, as it needs IT resources and must be harmonized with other pending or planned changes in the EHRs, and other interventions of the IT specialists. The children’s hospital of Basel, which is about to renew its IT infrastructure, started to implement SwissPedData in 2020, and other hospitals are planning to follow soon. Meeting with IT specialists and senior physician representatives of all collaborating hospitals are being planned. The central SPHN data centre and the IT staff of the five university hospitals, who receive substantial annual core funding from the SPHN initiative, will support the process. Given the hierarchical organization of the university hospitals, it was agreed that a top-down process with strong commitment from all directors of the Children’s clinics will be the most effective approach for implementation. The actual data exchange and access can be implemented as dedicated paediatric module on the SPHN infrastructure.

#### Scaling up and integrating other data sources

After harmonization of the data generated in university hospitals and other teaching hospitals, the SPHN plans to involve other healthcare providers in the process with the ultimate goal of connecting individual patient data from primary, secondary and tertiary care settings. Initial efforts to expand connectivity will focus on other hospitals, and networks of paediatricians and general practitioners providing primary healthcare. The idea of integrating data from primary care such as data on immunisations was repeatedly expressed and welcomed by participants of the meeting. However, such an extension will require dedicated funding.

#### Using the data

It is important that concrete research proposals and projects begin building on the harmonized dataset immediately, even though the quality of the data that will be available at the time of implementation will not be perfect. As demonstrated in PEDSnet, missingness of data, coding errors and bugs are most easily spotted when the data are being used to answer actual research questions, and concise projects give the opportunity to improve the data batch by batch [61]. As also shown in PEDSnet, first projects can demonstrate that improving data quality and shareability is a win-win for all stakeholders. Early use of the data for research can:

- boost awareness of new opportunities for improving healthcare afforded by the availability of large, standardized, national dataset among clinicians, researchers, and patient representatives;
- improve the willingness of local hospital directors to speed up implementation of the dataset in the EHR of their hospital as an instrument to assess and improve quality of healthcare delivered and boost their clinic’s research output;
- motivate physicians to enter data properly into the EHR, and to actively contribute to improving the dataset by providing constructive comments that will lead to improved versions of SwissPedData.

#### Securing sustainable funding

For the project to continue, it will need a central coordination centre, which can coordinate and facilitate the paediatric research collaboration. The tasks of this central body would include communication with the medical and IT representatives of the different children’s clinics, with the SPHN Data Coordination Centre, with international research partners and with funders, assistance with writing grant applications and obtaining ethical approval and the provision of standardized datasets for research. Because relevant aspects are covered by the SPHN data centre, the additional infrastructure required for a paediatric module can be relatively limited, but, as a minimum, will require a senior scientist to lead it, a coordinator with a strong research background, ideally in paediatric medicine, who communicates with clinicians, research partners and funding institutions, and another person specialized in data handling and information technology, who can work at the data level and communicate with IT specialists from local hospitals and with the SPHN data centre. The collaborating clinics will also need financial support for IT staff to deliver local data to in regular intervals or at request. The coordination centre can rely on the wider SPHN network for assistance including advice regarding ELSI aspects, the provision of templates for DTUA, and communication with stakeholders from federal and cantonal governments.

The following possibilities for securing future funding were discussed during the meeting:

- Participate in suitable calls for proposals from funding bodies at the national (e.g. next SPHN call and the Swiss National Science Foundation) or international level (such as EU funding or NIH) and in multinational consortia;
- Charge cost-covering fees for data delivery and other services provided by the SwissPedData coordination centre. These costs can be included in budgets of research grants from conventional funders, such as the Swiss National Science Foundation or smaller funding agencies;
- Build up collaborations with industry, for instance for post-marketing studies.

International collaboration will be essential for achieving necessary sample sizes for certain research projects, particularly those relating to rare diseases and for studies that draw added value from comparisons between different healthcare systems. Such collaboration will also increase the national and international visibility and recognition of SwissPedNet and improve chances of securing sustainable funding.

## Conclusions and outlook

Taken together, these actions should lay the foundations for a national paediatric LHS in Switzerland and help improve healthcare for children. Once implemented, SwissPedData can serve as core dataset on which further data layers could in future be added. Such data could include primary care data, immunisations, school examinations, administrative data (birth and mortality records, socio-economic and demographic information) or information on lifestyle factors and environmental exposures collected directly from families, for instance, through mobile devices or web-based applications. Furthermore, as children grow older, their electronic health records will capture information on long term health outcomes in adult life. Such extensions would greatly increase the scope of possible research and allow large-scale epidemiological investigations of children’s health, potentially over their life course and contribute to reducing disease burden in adult life.

## Data Availability

For data and materials relating to this document, please contact the corresponding author, Prof Claudia Kuehni claudia.kuehni@ispm.unibe.ch

## Abbreviations

**DTUA**: Data Transfer and Use agreement
**LHS**: Learning health system
**FSO**: Federal Statistical Office
**PEDSnet**: A multi-specialty network that conducts observational research and clinical trials across multiple children’s hospital health systems in the US
**SIB**: Swiss Institute of Bioinformatics
**SPHN**: Swiss Personalized Health Network
**SPAC**: Swiss Paediatric Airway Cohort - an observational research project in Switzerland, which uses data obtained during outpatient encounters in large paediatric hospitals
**SwissPedData**: “Harmonising the collection of health-related data and biospecimens in paediatric hospitals throughout Switzerland”, an infrastructure development project of the SPHN funded in 2017
**SwissPedNet**: Swiss Research Network of clinical Pediatric Hubs
**SBP**: Swiss Biobanking Platform
**FIRE**: Family medicine ICPC Research using Electronic medical records
